# Improving Visualization of Female Breast Cancer Survival Estimates: Analysis Using Interactive Mapping Reports

**DOI:** 10.2196/publichealth.8163

**Published:** 2018-05-03

**Authors:** Awatef Ahmed Ben Ramadan, Jeannette Jackson-Thompson, Chester Lee Schmaltz

**Affiliations:** ^1^ Missouri Cancer Registry and Research Center University of Missouri-Columbia Columbia, MO United States; ^2^ Department of Mathematics, Science and Informatics Penfield College Mercer University Atlanta, GA United States; ^3^ Department of Health Management and Informatics School of Medicine University of Missouri-Columbia Columbia, MO United States; ^4^ MU Informatics Institute University of Missouri-Columbia Columbia, MO United States

**Keywords:** survival, female breast cancer, Missouri, cancer registry

## Abstract

**Background:**

The Missouri Cancer Registry collects population-based cancer incidence data on Missouri residents diagnosed with reportable malignant neoplasms. The Missouri Cancer Registry wanted to produce data that would be of interest to lawmakers as well as public health officials at the legislative district level on breast cancer, the most common non-skin cancer among females.

**Objective:**

The aim was to measure and interactively visualize survival data of female breast cancer cases in the Missouri Cancer Registry.

**Methods:**

Female breast cancer data were linked to Missouri death records and the Social Security Death Index. Unlinked female breast cancer cases were crossmatched to the National Death Index. Female breast cancer cases in subcounty senate districts were geocoded using TIGER/Line shapefiles to identify their district. A database was created and analyzed in SEER*Stat. Senatorial district maps were created using US Census Bureau’s cartographic boundary files. The results were loaded with the cartographic data into InstantAtlas software to produce interactive mapping reports.

**Results:**

Female breast cancer survival profiles of 5-year cause-specific survival percentages and 95% confidence intervals, displayed in tables and interactive maps, were created for all 34 senatorial districts. The maps visualized survival data by age, race, stage, and grade at diagnosis for the period from 2004 through 2010.

**Conclusions:**

Linking cancer registry data to the National Death Index database improved accuracy of female breast cancer survival data in Missouri and this could positively impact cancer research and policy. The created survival mapping report could be very informative and usable by public health professionals, policy makers, at-risk women, and the public.

## Introduction

In the United States, it is estimated that 12% of women will be diagnosed with breast cancer at one stage of their lives [1]. Nationally, the estimated new cases of breast cancer were 14% of all new cancer cases and the estimated deaths from breast cancer were 7% of all cancer deaths in 2013 [2].

Traditionally, incidence and mortality rates have been presented in data tables, a format that is easily understood by epidemiologists and statisticians, but one that does not meet the needs of all potential users of the data. Data visualization is an alternative means of portraying the burden of breast cancer at various levels (eg, county, region, state).

There is a critical need to build accurate fact sheets in the form of interactive and dynamic map reports of the breast cancer burden at the substate level in Missouri. Several studies emphasize the efficiency and importance of matching National Death Index (NDI) data to cancer registry data to ensure high quality and accurate population-based cancer survival statistics [3-5]. We matched the registry breast cancer data to the Social Security Death Index (SSDI) and the NDI. This contribution will be significant because, with more complete data to analyze, we can accurately estimate survival data for the State of Missouri.

Numerous evidence-based studies have concluded that the use of geographic mapping software allows users to interact in a timely manner with the datasets and publish high-quality interactive reports [6-8]. The Web-based mapping systems’ contribution is significant because these systems will enable users to visualize cancer data easily, and users can share this data with contributors in fields related to the visualized cancer. Distribution of geospatial health data could help public health leaders and decision makers in designing, developing, and adopting effective and efficient strategies and programs to improve public health outcomes targeting specific subpopulations within geographical areas [6-8].

A study by Koenig et al [9] recognized the impact of the interactive mapping visualization of health data on the public health field and health care-related laws and decisions. The study spotted the need for more interaction between mapmakers and the mapping reports’ beneficiaries [9].

The Missouri General Assembly includes 34 senators, each representing one of Missouri’s 34 districts. Every senate district included an annual average population of approximately 90,000 female residents (176,000 total residents) between 2004 and 2010 (study period). Most of the districts included whole counties. In high population density areas, including the Kansas City metropolitan area, Saint Louis metropolitan area, and the city of Springfield, district limits do not follow county boundaries [10,11].

We aim to measure the survival proportions of female breast cancer cases in the Missouri Cancer Registry database and to further analyze these survival data by stage and grade at diagnosis, by race, by age, and by senatorial district in Missouri for the period from 2004 through 2010. We also aim to visualize the survival data by Missouri state senatorial district by creating interactive mapping reports.

## Methods

The study design was an observational longitudinal epidemiological study. The Missouri Cancer Registry and Research Center updated vital status of female breast cancer cases by linking with death records from the Missouri Department of Health and Senior Services and the SSDI [12]. We extracted female breast cancer cases (59,674 covering all years in the Missouri Cancer Registry database) without a known date and cause of death and submitted a formatted file containing required fields to the National Center for Health Statistics for NDI linkage [13]. The NDI staff returned the search results. We assessed the results to identify true matches. Partially matched records were reviewed manually using specific criteria (eg, possible typos, use of spouse’s social security number, change of surname, use of compound names in a different order, use of nicknames). We then updated the database with the linkage results.

The female breast cancer cases in counties split by senate districts were loaded into Esri’s ArcMap [14] with the Census Bureau’s TIGER/Line Shapefiles [15] to determine their district based on their latitude and longitude. For this project, we used the State Senate districts that were defined by the redistricting following Census 2010 [16].

A database was created in SEER*Stat, a statistical software package for analyzing cancer data [17]; this database included cases diagnosed from 2004 through 2010 in which the tumor was the first reportable in situ or malignant tumor diagnosed in the woman’s lifetime. This resulted in a total of 24,908 malignant cases for most of the survival calculations and an additional 5130 in situ cases included only in stage-specific survival calculations. The 5-year cause-specific survival proportions and their 95% confidence intervals were calculated for female breast cancer cases diagnosed from 2004 through 2010. Survival was measured in terms of cause-specific survival using the Surveillance, Epidemiology, and End Results (SEER) program’s cause-specific death classification recode as the endpoint [18]. The 5-year female breast cancer survival was calculated by age, race, stage, and grade for each senate district. To protect patient confidentiality, we suppressed cells with small numbers, employing a commonly used threshold of five or fewer cases [19].

The US Census Bureau’s cartographic boundary files were used to create maps showing 115 Missouri counties (including the City of St Louis—a county-equivalent entity) and 34 state senatorial districts [20]. Five-year survival statistics were loaded, along with cancer incidence and mortality data and the cartographic boundary files, into InstantAtlas software to produce interactive mapping reports that display our study’s results [21]. The interactive reports included maps, graphs, and tables for each county and Missouri senatorial district as well as for 20 regions formed by aggregating senate districts by county boundaries. The senate district grouped to county boundaries were created because mortality data was not available at the subcounty level.

The years of female breast cancer diagnoses we chose for this study were from January 1, 2004 through December 31, 2010, with survival calculated by including follow-up through December 31, 2011. When this project was started, 2011 was the most recent year with complete survival follow-up for female breast cancer cases. The case selection criteria we used for survival excluded cases diagnosed in 2011 because a relatively large number of cases diagnosed in that year may have been reported too late to be included in the death linkages or even too late to be included in the Missouri Cancer Registry database. The beginning year of the case selection criteria—2004—was chosen such that relatively stable estimates could be obtained for a wide variety of demographic groups of interest while still covering a relatively recent set of years (7 years total).

We classified female breast cancer cases as “early stage” if the stage at diagnosis was in situ or localized according to the Derived SEER Summary Stage 2000 field [22]; “late-stage” female breast cancer cases included regional and distant cases. Low-grade female breast cancer cases involved grades I and II; high-grade female breast cancer cases included grades III and IV.

## Results

The senatorial districts’ 5-year cause-specific survival proportions of female breast cancer were categorized, as shown in Tables 1-4, according to the following groupings: all malignant cases, cases younger than 50 years, cases 50 to 64 years, cases 65 years or older, white cases, African-American cases, early-stage (in situ and local) cases, late-stage (regional and distant) cases, low-grade cases, and high-grade cases. These tables include female breast cancer case counts and survival data for all 34 senatorial districts and Missouri and the 95% confidence intervals of the measured survival data for all the previously mentioned categories. Using these tables, the reader can compare every district to one another, as well as to the state’s survival proportion.

**Figure 1:**
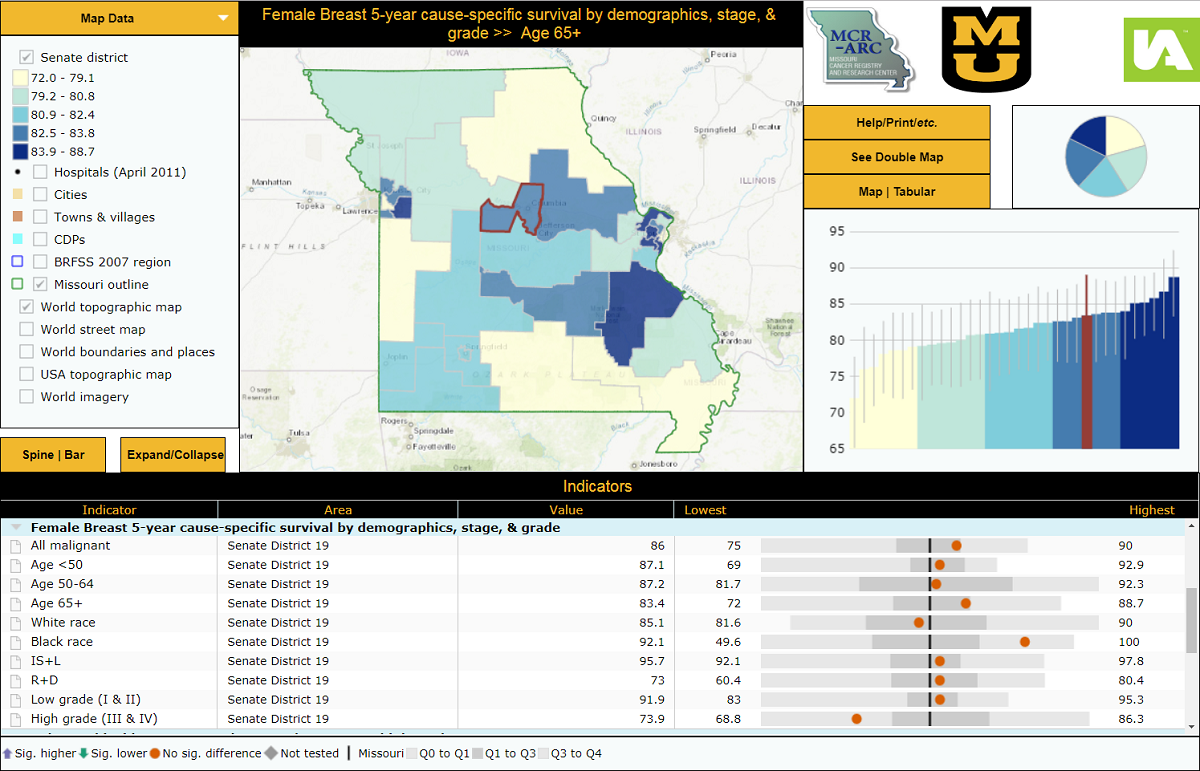
Area Profile Interactive Report Displaying FBC 5-Year Cause-specific Survival Data by Senatorial District [23].

**Figure 2:**
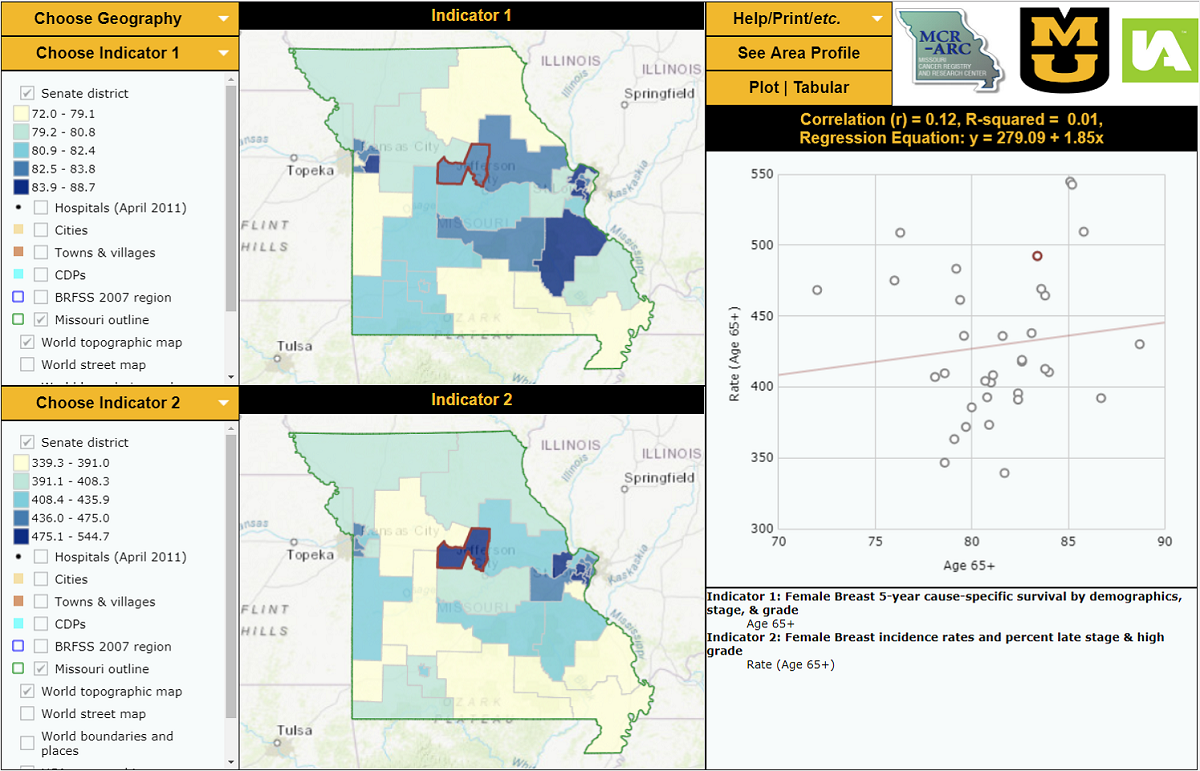
Double Map Interactive Report Displaying FBC 5-Year Cause-specific Survival Data by Senatorial District [24].

The reports we created displayed survival data results in two layouts: an “area profile” focused on displaying many indicators for one or a small number of selected districts along with results from statistical hypothesis testing (Figure 1) and a “double map” that displays two indicators simultaneously along with a district-level scatterplot (Figure 2). These reports include combined maps and statistical data. The area profile map displays a single map and presents many indicators for each senatorial district and compares each district’s results to the State of Missouri. The double map centers on assessing the statistical associations (correlation coefficient, *R*^2^, and the simple linear regression equation) among the chosen survival indicators. The screenshots displayed in Figures 1 and 2 show the final formats of the interactive mapping reports we built at the Missouri Cancer Registry and Research Center to display Missouri female breast cancer survival data along with other incidence and mortality data [23,24].

**Table 1:** Five-year cause-specific female breast cancer survival across different age groups by state senatorial district, Missouri, 2004-2010.

Senatorial district	<50 years		50-64 years		≥65 years	
	Cases, n	Survival %^a^ (95% CI)	Cases, n	Survival %^a^ (95% CI)	Cases, n	Survival %^a^ (95% CI)
1	181	91.6 (85.5-95.3)	302	90.7 (85.6-94.0)	428	85.8 (81.3-89.3)
2	192	92.9 (87.2-96.1)	224	89.9 (84.2-93.6)	198	79.2 (71.7-84.9)
3	128	77.2 (67.6-84.3)	251	88.8 (83.7-92.4)	272	84.0 (77.4-88.8)
4	163	89.5 (82.2-93.9)	300	86.9 (81.7-90.7)	435	76.3 (70.9-80.8)
5	167	81.8 (74.2-87.4)	230	86.3 (79.6-91.0)	231	76.0 (68.6-81.8)
6	160	85.6 (77.9-90.7)	234	86.6 (80.7-90.8)	314	81.0 (75.1-85.7)
7	147	86.0 (77.6-91.4)	269	86.9 (81.0-91.1)	264	83.8 (78.0-88.1)
8	181	87.2 (80.4-91.7)	261	86.5 (80.3-90.8)	208	86.7 (80.2-91.2)
9	158	69.0 (59.6-76.7)	273	81.8 (75.4-86.7)	288	72.0 (65.2-77.7)
10	147	90.5 (82.8-94.8)	261	86.2 (79.9-90.6)	267	83.8 (77.9-88.2)
11	130	84.7 (75.6-90.6)	240	84.4 (78.2-88.9)	326	82.6 (77.0-87.0)
12	152	91.1 (84.1-95.1)	254	84.7 (78.6-89.2)	300	80.8 (74.6-85.6)
13	207	85.3 (78.3-90.2)	290	84.0 (78.3-88.2)	356	79.6 (73.9-84.2)
14	212	80.6 (73.4-86.0)	319	87.0 (81.8-90.8)	283	83.1 (76.9-87.7)
15	240	92.5 (87.5-95.5)	368	90.6 (86.4-93.6)	403	85.1 (80.2-88.9)
16	139	87.5 (80.0-92.3)	237	84.3 (78.1-88.9)	306	82.6 (76.9-87.1)
17	173	85.6 (77.5-91.0)	258	92.3 (87.9-95.2)	268	83.6 (77.0-88.4)
18	155	85.4 (77.1-90.8)	259	83.4 (77.1-88.1)	388	78.1 (72.7-82.6)
19	167	87.1 (79.9-91.8)	238	87.2 (81.0-91.5)	206	83.4 (75.5-89.0)
20	155	90.6 (83.8-94.6)	251	86.4 (80.0-90.8)	260	82.4 (75.7-87.4)
21	137	82.7 (73.1-89.1)	250	84.9 (78.9-89.3)	296	80.0 (73.6-85.1)
22	138	87.0 (78.3-92.4)	215	90.7 (84.8-94.5)	184	80.9 (73.0-86.7)
23	181	90.2 (83.5-94.3)	304	91.2 (86.9-94.2)	267	88.7 (83.3-92.4)
24	188	91.0 (84.8-94.8)	371	91.0 (86.9-93.9)	448	85.2 (80.6-88.8)
25	142	82.9 (74.3-88.9)	274	81.7 (75.7-86.4)	327	79.1 (73.2-83.8)
26	204	88.4 (81.9-92.6)	338	89.5 (84.1-93.2)	327	79.4 (73.8-83.9)
27	151	86.6 (77.9-92.1)	257	84.8 (78.9-89.2)	318	79.7 (73.5-84.5)
28	144	84.5 (76.2-90.1)	294	85.5 (79.5-89.8)	352	82.4 (77.2-86.6)
29	117	85.0 (75.2-91.2)	250	90.7 (85.6-94.0)	349	81.1 (75.3-85.6)
30	145	82.0 (73.2-88.1)	232	85.8 (78.8-90.6)	341	81.6 (76.0-86.0)
31	146	87.3 (78.6-92.6)	253	87.6 (81.5-91.8)	293	78.6 (72.0-83.8)
32	155	77.2 (67.8-84.1)	245	86.7 (80.7-91.0)	287	81.7 (75.8-86.4)
33	137	88.2 (80.2-93.1)	231	83.8 (77.4-88.6)	273	78.6 (71.9-83.8)
34	157	87.5 (80.1-92.3)	252	85.3 (78.7-90.0)	264	80.7 (74.4-85.6)
Missouri	5496	86.1 (85.0-87.2)	9085	87.0 (86.1-87.8)	10,327	81.4 (80.4-82.3)

^a^Five-year cause-specific survival.

**Table 2:** Five-year cause-specific female breast cancer survival data among whites and African Americans by state senatorial district, Missouri, 2004-2010.

Senatorial District	White		African American	
	Cases, n	Survival %^a^ (95% CI)	Cases, n	Survival %^a^ (95% CI)
1	871	88.8 (86.0-91.1)	27	88.4 (68.2-96.1)
2	590	87.1 (83.6-89.9)	12	88.9 (43.3-98.4)
3	643	84.7 (81.1-87.8)	—^b^	—
4	578	85.4 (81.5-88.5)	302	76.4 (70.2-81.5)
5	248	85.8 (80.2-90.0)	370	78.6 (73.0-83.1)
6	691	84.6 (81.1-87.4)	11	58.3 (23.0-82.1)
7	536	87.6 (84.0-90.5)	133	77.7 (67.1-85.3)
8	618	86.9 (83.3-89.8)	25	77.1 (53.2-89.8)
9	292	81.6 (75.7-86.2)	418	70.4 (64.6-75.5)
10	645	86.1 (82.5-89.0)	26	91.8 (71.1-97.9)
11	654	83.5 (79.9-86.6)	33	84.7 (58.4-95.0)
12	698	84.5 (80.9-87.4)	—	—
13	489	85.4 (81.3-88.7)	352	77.6 (71.7-82.5)
14	368	89.1 (84.6-92.4)	435	79.6 (74.6-83.7)
15	966	89.3 (86.7-91.4)	14	68.4 (35.9-86.8)
16	661	84.7 (81.2-87.6)	12	67.3 (27.7-88.5)
17	673	87.8 (84.5-90.5)	16	75.7 (41.6-91.6)
18	769	81.8 (78.3-84.8)	28	67.5 (41.8-83.8)
19	549	85.1 (81.1-88.4)	42	92.1 (77.5-97.4)
20	662	85.8 (82.2-88.8)	—	—
21	661	82.7 (78.8-85.9)	18	65.6 (34.3-84.7)
22	525	86.0 (82.0-89.2)	—	—
23	722	90.0 (87.1-92.3)	24	85.2 (60.6-95.0)
24	897	89.0 (86.2-91.2)	72	80.8 (67.4-89.2)
25	688	81.6 (77.9-84.7)	49	67.4 (48.1-80.8)
26	840	85.5 (82.3-88.1)	7	100.0 (—)
27	704	83.7 (80.1-86.7)	20	49.6 (14.6-77.4)
28	778	83.9 (80.5-86.7)	—	—
29	706	85.3 (81.8-88.1)	—	—
30	698	83.3 (79.6-86.3)	10	56.4 (7.5-88.1)
31	671	84.1 (80.3-87.2)	12	69.1 (29.4-89.4)
32	675	82.2 (78.4-85.4)	—	—
33	636	82.4 (78.6-85.6)	—	—
34	637	84.5 (80.8-87.5)	19	72.8 (41.2-89.2)
Missouri	22,039	85.4 (84.8-85.9)	1607	76.8 (74.7-78.7)

^a^Five-year cause-specific survival.

^b^“—” indicates Survival statistics suppressed due to five or fewer cases.

**Table 3:** Five-year cause-specific female breast cancer survival data by stage at diagnosis and state senatorial district, Missouri, 2004-2010.

Senatorial district	All malignant cases (excludes in situ but includes unstaged cases)		Early stage (in situ & local)		Late stage (regional & distant)	
	Cases, n	Survival %^a^ (95% CI)	Cases, n	Survival %^a^ (95% CI)	Cases, n	Survival %^a^ (95% CI)
1	911	88.6 (85.9-90.9)	763	96.8 (94.7-98.1)	328	77.5 (71.3-82.6)
2	614	87.3 (83.9-90.1)	497	97.5 (95.4-98.6)	244	75.5 (68.5-81.2)
3	651	84.4 (80.7-87.4)	467	96.1 (93.2-97.8)	267	72.3 (65.3-78.2)
4	898	82.5 (79.2-85.2)	703	95.4 (93.0-97.0)	365	68.6 (62.5-73.9)
5	628	81.5 (77.6-84.8)	459	95.1 (92.0-97.0)	274	66.3 (58.9-72.6)
6	708	84.0 (80.5-86.9)	637	94.7 (92.2-96.4)	232	70.7 (63.5-76.7)
7	680	85.4 (81.9-88.3)	562	95.9 (93.3-97.5)	283	74.4 (67.8-79.8)
8	650	86.7 (83.2-89.5)	555	95.5 (92.8-97.2)	254	78.0 (71.0-83.5)
9	719	75.0 (70.9-78.6)	550	92.1 (88.7-94.5)	323	60.4 (53.5-66.6)
10	675	86.2 (82.7-89.0)	536	95.7 (93.1-97.3)	251	76.4 (68.9-82.2)
11	696	83.6 (80.1-86.6)	547	94.6 (91.8-96.4)	272	74.1 (67.5-79.6)
12	706	84.5 (80.9-87.4)	552	95.3 (92.6-97.0)	269	71.7 (64.5-77.8)
13	853	82.6 (79.3-85.4)	662	94.6 (92.1-96.3)	355	69.8 (63.7-75.1)
14	814	83.9 (80.6-86.7)	627	96.2 (93.7-97.7)	362	71.7 (65.8-76.8)
15	1011	89.0 (86.4-91.1)	889	96.3 (94.3-97.6)	344	78.3 (72.6-82.9)
16	682	84.3 (80.8-87.2)	529	95.2 (92.5-96.9)	256	74.5 (67.7-80.2)
17	699	87.4 (84.1-90.1)	549	97.7 (95.5-98.9)	261	73.4 (66.1-79.3)
18	802	81.3 (77.8-84.3)	608	94.9 (92.2-96.7)	309	67.7 (61.1-73.4)
19	611	86.0 (82.3-89.0)	497	95.7 (92.6-97.5)	230	73.0 (65.5-79.0)
20	666	85.9 (82.3-88.8)	576	95.6 (93.0-97.3)	240	77.1 (70.0-82.7)
21	683	82.3 (78.5-85.5)	486	95.8 (92.8-97.6)	296	68.7 (61.7-74.7)
22	537	86.4 (82.5-89.5)	441	95.4 (92.4-97.2)	191	76.2 (68.1-82.6)
23	752	90.0 (87.2-92.2)	616	97.8 (95.6-98.9)	293	80.4 (74.5-85.1)
24	1007	88.5 (85.9-90.7)	854	96.5 (94.5-97.8)	343	77.2 (71.3-81.9)
25	743	80.8 (77.2-83.9)	516	94.7 (92.0-96.6)	291	66.4 (59.4-72.5)
26	869	85.4 (82.3-88.1)	733	94.9 (92.4-96.6)	302	74.1 (67.6-79.5)
27	726	83.0 (79.4-86.0)	549	94.6 (91.7-96.5)	272	69.6 (62.3-75.7)
28	790	84.0 (80.6-86.8)	603	95.7 (93.1-97.4)	301	73.3 (67.0-78.6)
29	716	85.2 (81.8-88.0)	571	96.7 (94.4-98.1)	259	71.5 (63.8-77.9)
30	718	83.0 (79.4-86.1)	611	94.2 (91.2-96.2)	242	69.5 (62.1-75.7)
31	692	83.8 (80.1-86.9)	555	94.4 (91.4-96.4)	256	75.0 (67.6-80.9)
32	687	82.4 (78.7-85.6)	550	94.7 (91.8-96.5)	250	67.2 (59.5-73.8)
33	641	82.6 (78.7-85.7)	542	93.0 (89.6-95.3)	205	69.7 (62.0-76.2)
34	673	84.1 (80.5-87.1)	543	96.0 (93.4-97.6)	251	70.8 (63.3-76.9)
Missouri	24,908	84.5 (84.0-85.0)	19,935	95.5 (95.1-95.8)	9471	72.3 (71.2-73.4)

^a^Five-year cause-specific survival.

**Table 4:** Five-year cause-specific female breast cancer survival data for low- and high-grade cases, Missouri, 2004-2010.

Senatorial district	Low-grade (I & II)		High-grade (III & IV)	
	Cases, n	Survival %^a^ (95% CI)	Cases, n	Survival %^a^ (95% CI)
1	576	93.8 (90.6-95.9)	232	84.0 (77.9-88.6)
2	395	94.2 (90.8-96.4)	184	81.5 (74.0-87.0)
3	334	93.2 (89.1-95.8)	239	75.9 (68.5-81.8)
4	510	90.5 (86.8-93.2)	321	75.8 (69.5-80.9)
5	309	87.6 (82.3-91.3)	273	76.8 (70.3-82.1)
6	431	90.8 (86.9-93.6)	205	77.3 (70.0-83.1)
7	420	93.1 (89.4-95.5)	208	76.9 (69.3-82.8)
8	405	91.9 (87.6-94.8)	200	77.6 (70.2-83.5)
9	416	83.0 (77.8-87.1)	247	68.8 (61.5-75.0)
10	444	91.1 (86.8-94.0)	172	79.7 (71.7-85.7)
11	431	90.3 (86.6-93.0)	202	75.7 (68.0-81.8)
12	443	91.2 (87.5-93.9)	203	76.0 (67.5-82.6)
13	494	89.9 (86.1-92.7)	296	71.3 (64.8-76.8)
14	433	91.9 (88.1-94.5)	325	77.7 (71.7-82.5)
15	605	95.2 (92.5-96.9)	308	81.7 (75.7-86.3)
16	383	90.0 (85.8-93.0)	240	80.5 (73.9-85.5)
17	454	91.5 (87.9-94.1)	202	82.4 (74.4-88.2)
18	474	90.2 (86.3-93.0)	233	75.7 (68.5-81.5)
19	378	91.9 (87.6-94.8)	197	73.9 (65.8-80.3)
20	377	95.3 (92.0-97.3)	257	77.7 (70.9-83.1)
21	427	89.6 (85.4-92.7)	199	76.8 (69.1-82.8)
22	290	94.4 (89.8-96.9)	200	77.6 (69.7-83.7)
23	501	92.3 (88.9-94.8)	220	86.3 (80.2-90.7)
24	634	92.8 (89.8-95.0)	292	85.3 (79.8-89.4)
25	386	89.1 (84.4-92.4)	253	73.7 (66.9-79.4)
26	539	91.9 (88.4-94.3)	271	77.8 (71.0-83.2)
27	402	90.8 (86.7-93.7)	250	76.8 (69.7-82.4)
28	442	91.8 (88.0-94.4)	289	81.9 (76.0-86.5)
29	428	91.5 (87.4-94.3)	243	81.2 (74.7-86.3)
30	402	91.4 (87.4-94.1)	281	77.0 (70.1-82.6)
31	393	90.9 (86.6-93.9)	235	80.9 (73.6-86.3)
32	368	92.8 (88.6-95.5)	277	72.1 (65.1-78.0)
33	358	92.0 (87.8-94.8)	240	75.4 (68.4-81.1)
34	463	89.4 (85.4-92.3)	159	78.7 (69.2-85.5)
Missouri	14,745	91.4 (90.8-92.0)	8153	77.8 (76.7-78.8)

^a^Five-year cause-specific survival.

## Discussion

### Principal Findings

The Missouri Cancer Registry needs to measure female breast cancer survival proportions to be able to evaluate the impact of Missouri’s breast cancer control program and the burden of female breast cancer in Missouri. The measured and visualized survival data will transform our registry from being an incidence registry to becoming a survival registry for breast cancer.

Survival data mirrors female breast cancer prediction in a specific period [25]. We used the Missouri Cancer Registry records because Missouri Cancer Registry is a nationally recognized, population-based registry with data that originates from diverse sources including hospitals, ambulatory surgical centers, freestanding cancer treatment centers, pathology laboratories, long-term care facilities, and physician offices. It also contains cases obtained through case-sharing agreements with 19 states. The Missouri Cancer Registry data undergo a strict quality control process and the data are evaluated following specific national measures [26]. Several studies have revealed the significance of linking NDI data to a central cancer registry’s data to obtain more accurate population-based cancer survival data [3-5].

From this study’s results, as shown in Tables 1-4, we can create female breast cancer survival profiles for the 34 Missouri senate districts. By creating these profiles, we can compare each district’s results to the state and to other districts’ results and give more detailed information to public health practitioners and decision makers about female breast cancer in their district.

### Mapping Reports

Cancer incidence and mortality data have traditionally been presented in tabular and descriptive statistics formats; these are easily understood by health professionals with specific knowledge and experience in statistics and epidemiology. At the Missouri Cancer Registry and Research Center, we strive to present our data in formats that meet the needs of a wide range of potential data users. That is why we chose to combine our survival data with geographical data to produce interactive mapping reports at the Missouri senate district level. InstantAtlas is an interactive, internet-based mapping tool licensed to the Missouri Cancer Registry and Research Center that allows users to visually display data gathered from the registry and other databases. Use of interactive data visualization and mapping software allows users to interact with the datasets. We built two interactive mapping reports that include our senate district-level female breast cancer survival data [23,24]. The two maps, the area profile map and the double map, have not yet been published on the Missouri Cancer Registry and Research Center’s website. The area profile report shows a single map and focuses on displaying many indicators for a selected state senate district and compares the district’s findings to other districts and to Missouri. The double map focuses on exploring the relationships between selected indicators; it displays two indicators simultaneously along with a scatterplot or a table.

The InstantAtlas reports can facilitate communication between collaborators from different fields related to breast cancer, enhance female breast cancer research and policy, and inform public health professionals and policy makers. These maps can be used as educational tools at the community level for women at risk and the public about the distribution of female breast cancer in Missouri by age, race, stage and grade at diagnosis, and by senatorial district. These data could be used as a knowledge base at Missouri oncology facilities to assess management plan decisions taken by providers and by female breast cancer cases.

### Study Challenges and Limitations

During the matching processes, some cases did not have a social security number, which is the best available unique identifier. Also, some identifiers, such as date of birth and last and/or first name, showed differences when the NDI database and the registry database were compared, possibly due to data entry errors or changed last name. Such cases were manually reviewed. Manual review of all partial matches was done by more than one Missouri Cancer Registry and Research Center staff member, including at least one certified tumor registrar, to reduce possible mistakes.

Survival was measured using cause-specific survival rather than relative survival (another common net measure of survival) to avoid the need of having detailed population lifetables by senatorial district. Potential disadvantages of using cause-specific survival is that, unlike relative survival, it relies on additionally having the cause of death rather than just the fact and date of death and on accurate coding of the cause of death [18]. To decrease the number of known decedents with unknown cause of death in the Missouri Cancer Registry database, these cases were included in the NDI linkage to try to obtain their cause of death. To lessen the impact of miscoded cause of death (eg, a breast cancer death being misattributed to the location of a metastatic site), the data used here was defined “breast cancer death” according to the SEER cause-specific death classification recode variable [18]. It should be noted that this will miss indirect deaths originating from a diagnosis of breast cancer, such as toxic effects of chemotherapy. Moreover, the use of this death classification variable is limited to first primary tumors only as used in these analyses and cannot be used to analyze second and subsequent tumors.

Due to aggregating the cases to areal units, this study is subject to the modifiable areal unit problem [27]. State senate areal units were selected for this project because they would be relevant to policy makers making decisions at the senate district level and to constituents within those districts. It should be noted that the modifiable areal unit problem implies that differing conclusions can potentially be drawn from the same data had different areal units been used.

The survival rates presented in these mapping reports are the observed percentages rather than rates that have been spatially smoothed. Observed percentages may be more directly interpretable and relevant to the residents of each of the individual senate districts; however, observed percentages have the disadvantage of being less stable and more prone to spurious high and low values than spatially smoothed survival rates. This instability is mitigated somewhat by the fact that, with the notable exception of African Americans in many districts, survival was calculated with fairly large sample sizes: always more than 100 and generally at least 200 or more.

For the selected cases, only approximately 1% had the district imputed. Due to the relatively small number of cases, a sensitivity analysis was not performed.

### Future Directions

In the future, by combining mortality and incidence data in the survival profiles, we will be able to inform every district’s decision makers about the full picture of female breast cancer burden by district and we could help them assess female breast cancer interventions and policies on geographical bases. Due to small sample sizes, we do not have county-level results from the Behavioral Risk Factor Surveillance System, a state-based health survey that annually gathers data on health events, behaviors, preventive practices, and access to health care. A similar Behavioral Risk Factor Surveillance System-based survey known as the “County-Level Study” has been conducted at the county level in Missouri [28]. In the future, we hope to combine these results with female breast cancer survival data and create InstantAtlas mapping reports at the senatorial district level that include survival and other measured contextual indicators (eg *,* demographic, environment, and socioeconomic), similar to the currently published county-level maps. This kind of mapping report could be used to explore the relationship between female breast cancer and other measured contextual indicators all over Missouri.

In this paper, we measured 5-year cause-specific survival proportions of female breast cancer for the 34 senate districts in Missouri. In the future, we will consider the feasibility of measuring the same data for all 163 Missouri legislative districts [29,30]. We will also consider measuring 5-year cause-specific survival for other screening-amenable cancers (eg, colorectal cancers) and for cancers that impact many residents (eg, lung cancer).

Before we publish senate district maps on our website, we aim to test the usability of the survival maps using a pilot sample of actual users, similar to one we conducted with our previously published maps [31], in order to make them more user friendly.

### Conclusions

Net measures of survival factor out other causes of death and are useful from a policy-based perspective. These measures enable comparisons of cancer survival across geographical regions and between groups of patients without differences in background mortality rates of other causes impacting the results.

Cancer registry data are very rich and can be used in the exploration of many scientific theories and models. Registry data are a valuable source for survival data on breast cancer by race, age, and stage at diagnosis. Using cancer registry data supplemented by SSDI and NDI information will be beneficial and can improve accuracy of breast cancer survival data by age, stage, or race, as well as by geographic area (counties and senatorial districts).

## Abbreviations

NDI: National Death Index
SEER: Surveillance, Epidemiology, and End Results
SSDI: Social Security Death Index
